# Transmission of Information in Neoplasia by Extracellular Vesicles

**DOI:** 10.1155/2015/289567

**Published:** 2015-11-30

**Authors:** Aurelio Lorico, Denis Corbeil, John M. Pawelek, Riccardo Alessandro

**Affiliations:** ^1^Cancer Research Center, Roseman University College of Medicine, Las Vegas, NV 89135, USA; ^2^Tissue Engineering Laboratories (BIOTEC) and DFG Research Center and Cluster of Excellence for Regenerative Therapies Dresden, Technische Universität Dresden, Tatzberg 47-49, 01307 Dresden, Germany; ^3^Yale University, New Haven, CT, USA; ^4^Department of Biopathology and Medical Biotechnology, University of Palermo, Via Divisi 83, 90133 Palermo, Italy

Paracrine interactions among neoplastic and nonneoplastic cells in the immediate tumor microenvironment are important for tumor growth and metastatic spreading. Most of the studies in the past decade addressing these cellular interactions have focused on tumor cell-derived soluble molecules. Recently, these studies and interest have shifted to nanosized extracellular vesicles (EVs) and especially ectosome and exosome-associated molecules [1]. They contain not only proteins, but also lipids, mRNA, and microRNA [1], which can regulate gene expression in their target cells in a much more pleiotropic manner [1]. While exosomes originate by a sequential process of inward budding of late endosomes, producing multivesicular bodies (MVBs), followed by release of internal microvesicles into the microenvironment by fusion of the MVBs with the plasma membrane [1, 2], ectosomes bud from plasma membrane, particularly from plasma membrane protrusions (e.g., microvilli) [1]. However, difficulties in obtaining homogeneous exosomal and ectosomal preparations result in incomplete understanding of their formation, composition, and functions [3]. Trafficking of biological materials across cellular membranes is part of any normal cell homeostasis, to maintain proper compartmentalization of important molecules. The physiological functions of EVs are extremely diverse (e.g., cell-cell communication, cellular differentiation, immunity, and inflammation) [4–8]. However, in pathological states, such as cancer, aberrant activity of the export machinery results in expulsion of a number of key proteins and microRNA that modify the tumor microenvironment, in turn stimulating the release of EVs from stromal and immune-competent cells. In cancers, such vehicles might play a role in carcinogenesis and disease progression and promote formation of the premetastatic niche [9–11]. In the present special issue on transmission of information in neoplasia by extracellular vesicles, review articles and research paper illustrate the relevance of EVs to cancer growth and metastasis.

C. Soekmadji and C. C. Nelson discuss the current available literature about the role of EV-mediated drug resistance in cancers, with a particular focus on advanced prostate cancer. Presence of multidrug resistance proteins on the EV membrane, enrichment of ceramide, and sequestration of anticancer drugs are all potential EV-mediated mechanisms to limit the bioavailability or efficacy of anticancer agents.

T. M. Green et al. review the effects of EVs released by breast cancer cells through transfer of mRNA, microRNA, and proteins to different recipient cells within the tumor microenvironment, both in an autocrine and in a paracrine manner, which have a significant impact on signaling pathways, mRNA transcription, and protein expression. The authors point to proteins and microRNA identified within breast cancer-released EVs that give insight as to the nature and severity of the disease and could serve as possible diagnostic markers. Emphasis is also placed on multiple mechanisms by which breast cancer cells avoid immune system recognition through EVs, such as secretion of immunosuppressive proteins, inhibition of NK cell proliferation, or a decrease in T cell cytotoxicity. Finally, potential benefits deriving from the use of EVs as a vehicle for delivery of anti-breast cancer drugs are discussed.

T. Sun et al. point to exosome-derived noncoding RNA and in particular microRNA as important molecules in lung cancer biology, facilitating lung cancer growth and metastasis. Both the EV field and the microRNA fields being in their infancy, it is conceivable that many biological mechanisms crucial for lung carcinogenesis and progression have yet to be discovered.

Z. Qian et al. review the current literature in the field of EV-mediated changes in the epigenetics of cancer microenvironment. Some of the proteins and nucleic acids transmitted through EVs to recipient cells participate in DNA methylation, histone modification, and posttranscriptional regulation of RNA. They may be responsible for altering expression of oncogenes and tumor suppressor genes in recipient cells.

S. Raimondo et al. examine the role of EVs released from cells of hematological malignancies during cancer progression, mainly focusing on the specific microenvironment of hematological diseases, that is, the bone marrow. Blast cells from acute myeloid leukemia and chronic myeloid leukemia are able to initiate an exosome-mediated cell-cell communication with bone marrow stromal cells, thus affecting angiogenesis and microenvironmental niche. Furthermore, examples of the acquisition of EV-mediated chemotherapeutic resistance are illustrated for multiple myeloma and B-cell lymphoma.

In their review article, G. Schiera et al. summarize the possible physiological roles of EVs in the nervous system, where cell-cell communication is determinant in neuron and glia differentiation as well as in the formation of the blood-brain barrier. They further discuss the involvement of EVs in the horizontal transfer of information for brain pathologies such as Alzheimer's disease, Parkinson's disease, and amyotrophic lateral sclerosis.

In the article entitled “High LIN28A Expressing Ovarian Cancer Cells Secrete Exosomes That Induce Invasion and Migration in HEK293 Cells,” the authors present the results of a study on IGROV1 ovarian cancer cells expressing high levels of LIN28, a stem cell marker and emergent oncogenic driver. Although LIN28 was not present in exosomes released by cultures of IGROV1 cells, addition of exosomes to target cells resulted in changes in expression of genes associated with epithelial-mesenchymal transition and in a more aggressive cell behavior.

Overall, the current issue of this journal highlights the contribution of EVs to tumorigenesis and formation of metastases and points to several potential strategies to effectively utilize EVs for breast cancer diagnosis, prognosis, and therapy.

*Aurelio Lorico* *Denis Corbeil* *John M. Pawelek* *Riccardo Alessandro*
